# A Colorimetric Sensor for Qualitative Discrimination and Quantitative Detection of Volatile Amines

**DOI:** 10.3390/s100706463

**Published:** 2010-06-30

**Authors:** Zhonglin Tang, Jianhua Yang, Junyun Yu, Bo Cui

**Affiliations:** School of Automation, Northwestern Polytechnical University, Xi’an 710072, China; E-Mails: leejin_111@sohu.com (Z.L.T.); autocad1998@163.com (J.Y.); cuibo@nwpu.edu.cn (B.C.)

**Keywords:** colorimetric sensor array, dyes, amine detection, color spaces

## Abstract

We have developed a novel colorimetric sensor based on a digital camera and white LED illumination. Colorimetric sensor arrays (CSAs) were made from a set of six chemically responsive dyes impregnated on an inert substrate plate by solution casting. Six common amine aqueous solutions, including dimethylamine, triethylamine, diisopropylamine, aniline, cyclohexylamine, and pyridine vaporized at 25 °C and six health-related trimethylamine (TMA) concentrations including 170 ppm, 51 ppm, 8 ppm, 2 ppm, 125 ppb and 50 ppb were analyzed by the sensor to test its ability for the qualitative discrimination and quantitative detection of volatile amines. We extracted the feature vectors of the CSA's response to the analytes from a fusional color space, which was obtained by conducting a joint search algorithm of sequential forward selection and sequential backward selection (SFS&SBS) based on the linear discriminant criteria (LDC) in a mixed color space composed of six common color spaces. The principle component analysis (PCA) followed by the hierarchical cluser analysis (HCA) were utilized to discriminate 12 analytes. Results showed that the colorimetric sensor grouped the six amine vapors and five TMA concentrations correctly, while TMA concentrations of 125 ppb and 50 ppb were indiscriminable from each other. The limitation of detection (LOD) of the sensor for TMA was found to be lower than 50 ppb. The CSAs were reusable for TMA concentrations below 8 ppm.

## 1. Introduction

Volatile amines are by-products of rapidly growing cells and commonly produced by organic decomposition. Their existence and concentrations are considered as quality indicators of foods [1,2] and biomarkers in metabolic diseases [3,4]. Traditional instrumental analysis methods such as gas chromatography (GC) and mass spectrometry (MS) play important roles in amine identification and detection [5,6], but they are usually expensive, time consuming, and can be operated only by trained personnel. Gas sensors (also known as electronic noses), developed as additional supplement to or even competitive substitute for the traditional analytical methods in gas identification and detection, have recently received increasing attention and have been quickly improving [7]. Among the different types of gas sensors, metal oxide sensors (MOS) are the most widely studied in volatile amine detection [8–11]. In response to gaseous analytes, MOS and other common gas sensors, such as the conducting polymer (CP) and the quartz crystal microbalance (QCM), generate unexceptionally a one-dimensional electrical signal, which reflects only one aspect of the properties of analyte molecules. For example, MOS probes and reflects the oxidation/reduction property of client molecules by resistance change; QCM probes and reflects the mass of client molecules by frequency change. Volatile amines span a wide range of physical and chemical properties. However, those amines differing significantly may appear similar for certain given properties. For example, cyclohexylamine and triethylamine share close molecular mass, but disagree in molecular structures, shapes, sizes, electronic and chemical properties. Thus, it remains a challenge for electrical signal sensors such as MOS or CP to identify effectively multiple amines and to differentiate among various amine concentrations at the same time.

The CSA of a colorimetric gas sensor shows a unique pattern of color change after exposure to some analytes, which is seen as the analytes’ “color fingerprint” [12]. When a dye on the CSA absorbs and reacts with gas molecules, their frontier molecular orbits interact with each other, which leads to shifts of the electronic transition energy in the reaction products and shows a color change under visible lighting [13]. This kind of absorption and reaction is essentially a chemical process, which, for a given sensitive dye, is defined by the comprehensive properties of the analyte molecules, including their electronic and chemical properties, molecular structures and stereo shapes. When imaged with common color imaging equipment such as a digital camera, there are three signal channels (red, green, blue) for each pixel on the CSA to carry the comprehensive information of the molecular properties of gaseous analytes, resulting a more powerful gas identification and detection capability than electrical signal gas sensors [14,15]. To date, the CSA has been monitored mainly by a flatbed scanner, and its color change patterns are unexceptionally extracted from the RGB color space. Since portability is important for gas sensors, and the three color channels in RGB color space are correlative [16] and may not be the best features for gas identification, we report herein a colorimetric gas sensor with LED lighting and digital camera imaging. In the experiments, the gas sensor was tested with six volatile amines comprising branched and cyclic structures for qualitative discrimination capability, and with six concentrations of TMA for quantitative detection capability and sensitivity. To improve the sensor’s capability of sample discrimination, a fusion of different color spaces was performed for response feature extraction. For the fusion of different color spaces, the images sampled in RGB color space by the digital camera were at first transformed to other five common color spaces. Then a SFS&SBS search algorithm based on LDC was conducted to pick out those color channels which showed the best sample discrimination ability in the six color spaces. The response feature vectors in the fusional color space were at first compressed by the PCA and then grouped by the HCA. The method introduced herein improved obviously the sample discrimination ability of the colorimetric senor, which grouped amines with different molecular structures correctly in the experiment. With improved lighting and imaging equipment, the sensor was very sensitive to TMA, with a LOD lower than 50 ppb, and showed sound reproducibility for a TMA concentration of 8 ppm.

## 2. Experiments

### 2.1. CSAs Preparation

**Figure 1.**
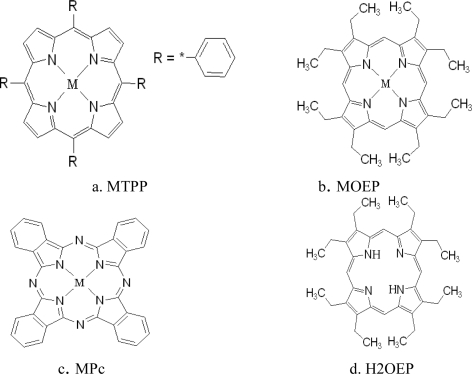
The molecular structures of the chemically sensitive dyes.

A CSA has a group of spots composed of different chemically sensitive dyes impregnated on an inert substrate plate. In this study, six kinds of dyes were chosen for amine discrimination and detection, including tetraphenylporphine zinc(II) (ZnTPP), octaethylporphine zinc(II) (ZnOEP), zinc phthalocyanine (ZnPc), tetraphenylporphine cobalt(II) (CoTPP), octaethylporphine cobalt(II) (CoOEP), and octaethylporphine (H2OEP). Their molecular structures are shown in Figure 1, where M means Zn or Co ions.

As shown in Figure 1, all dyes have a coordinate center for coordination reactions with ligand amine molecules and these reaction centers are coupled to intense chromophores (P: porphyrin or Pc: phthalocyanine) with different functional groups (TP: tetraphenyl- or OE: octaethyl-). Coordination reaction is a strong chemical reaction rather than a weak physical absorption. An amine molecule can be strongly bound to a dye center and the interaction is manifested as obvious color change on the chromophore. The change degree is modulated by the functional groups and the central ions simultaneously. These dyes make up the selective and cross sensitive array elements on the CSA.

**Figure 2.**
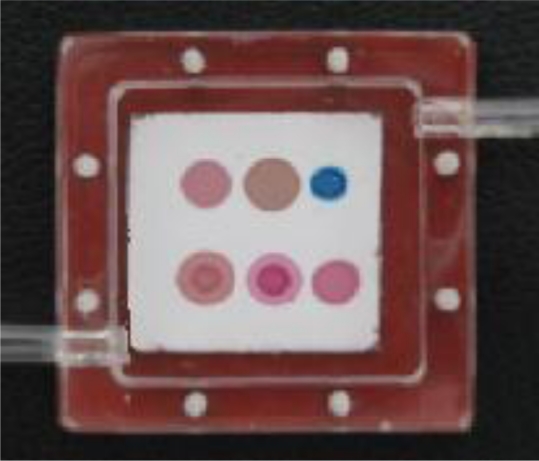
CSA. The dye spots are ZnTPP, ZnOEP, ZnPc, CoTPP, CoOEP and H2OEP, clockwise and sequentially from the top left corner, numbered from 1 to 6. The cartridge has an inner size of 30 × 30 × 10 mm^3^.

All the dyes were purchased from the Aldrich Company and used as received. Solutions of 2 mg/mL were made in chloroform and then cast on C2 reverse phase silica gel plates (the inert substrate, with size of about 25x20 mm^2^) with a 2 μL micro-syringe to form dye spots with a radius of about 2–3 mm. Arrays with six sensitive elements were fabricated. Finished CSAs were packaged in an optically clear and hermitically sealed cartridge of polymethyl methacrylate filled with pure N_2_, as shown in Figure 2, to prevent contamination during storage.

### 2.2. Colorimetric Sensor Construction

The colorimetric sensor reported here was composed of a digital camera, an illumination chamber, a CSA and a host PC. The digital camera was a Canon SX100, which supports the sRGB color standards and was driven by a personal computer through a USB interface. The illumination chamber had a bowl-like inner surface coated with a thin layer of diffuse reflecting material. LEDs with color temperatures of 3,000–3,500K were used as the lighting sources and were powered by a constant current source of 0.1% ripple. The light of the LEDs was first scattered by a scattering plate and then reflected back from the inner diffuse surface of the illumination chamber upon the CSA’s transparent surface, thus eliminating the specular reflection and ensuring the illumination spatially uniform. A blank C2 reverse phase silica gel plate was used for the white balance adjusting of the camera and was tested to evaluate the time stability and the spatial uniformity of the system. For time stability, the system was tested under room temperature of 24–28 °C continually for 20 minutes each time, three times each day and three days total. Ninety images were taken with a sample interval of two minutes. The mean RGB values of each image were then calculated and the results showed that the maximum vibrating amplitudes for the R, G and B color channels were within ±0.6 color units. A further test on nine NCS standard colors showed that the system’s time stability performance was consistent for different colors. For spatial uniformity, the blank substrate was uniformly divided into six sub-areas of two rows and three columns, the results showed that for the 90 images the mean RGB differences among the sub-areas were within 1 color unit, which might mainly come from the color variation on the substrate surface.

### 2.3. Experimental Setup

Seven 30 wt% (mass percentage) analytical reagent (AR) grade aqueous amine solutions were chosen to generate amine vapors, including dimethylamine, triethylamine, diisopropylamine, and TMA, which have branched molecular structures, and aniline, cyclohexylamine, and pyridine, which have cyclic molecular structures. For qualitative discrimination, amine vapors were generated by bubbling pure N_2_ at 200 mL/min through sealed 25 °C constant temperature bottles half filled with some amine aqueous solution, except for TMA, and collecting the headspace vapors; For quantitative detection, six TMA concentrations, including 170 ppm, 51 ppm, 8 ppm, 2 ppm, 125 ppb and 50 ppb, were generated by vaporizing a given quantity of TMA aqueous solution in a Teflon gas bag filled with 4 liters of pure N_2_ at a room temperature of 24–28 °C. According to the National Advisory Committee for Acute Exposure Guideline Levels for Hazardous Substances (NAC/AEGL Committee), for an 8-hour exposure time, TMA concentrations of 170 ppm, 51 ppm and 8 ppm are lethal, disabling, and non-disabling, respectively. TMA concentration of 125 ppb is set as the threshold of odor pollution in China.

**Figure 3.**
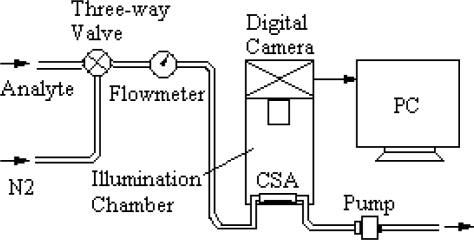
Schematic of colorimetric sensor for amines discrimination and detection

Schematics of the colorimetric sensor for amines discrimination and detection are shown in Figure 3. The analytes included the six amine headspace vapors and the six TMA concentrations. Images of the CSA both prior to and after exposure to analytes are collected at specified time intervals during the sensing experiments.

The gas flow rate in the system was set to 200 mL/min. The imaging interval was set to 1 minute. To begin an experiment, we first turned the three-way valve to the N_2_ channel to wash the gas lines for 5 minutes, then inserted the cartridge into the fixture on the illumination chamber and connected its inlet and outlet pipes to the system’s gas lines. For qualitative discrimination, three CSA images were sampled at first in a N_2_ stream and followed by 12 CSA images in an analyte stream; For quantitative detection, five CSA images were sampled first in a N_2_ stream and followed by five CSA images in an analyte stream. Ten images were sampled after that in N_2_ again for determination of the response reproducibility of the CSA on different TMA concentrations. For those TMA concentrations at which the CSA can respond reproducibly, the quantitative detection trials were conducted for three recycles. Five replicate trials were conducted for each analyte on different CSAs. For a total of 12 analytes, 60 image sequences with different frame numbers were obtained. For each image sequence of the 60 trials, the first five images of the CSA sampled immediately after exposure to the analyte were taken for feature extraction and sample discrimination.

## 3. Results and Discussion

### 3.1. CSA’s Color Response to Analytes

**Figure 4.**
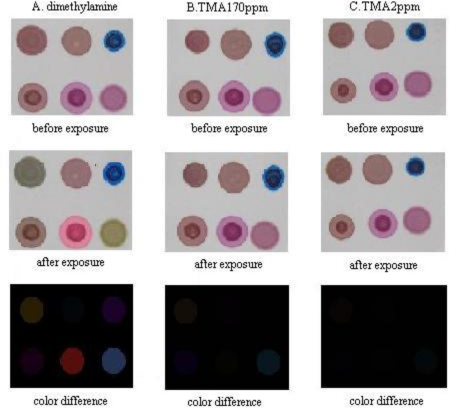
CSA’s images before and after exposure for 5 minutes to: (A) dimethylamine; (B) 170 ppm of TMA; and (C) 2 ppm of TMA. The color difference images reflect the mean color change of each dye on the CSA.

The CSA changes color after exposure to amines, as shown in Figure 4, where three CSA images before and after exposure for five minutes to dimethylamine, 170 ppm of TMA and 2 ppm of TMA are presented. The color difference images reflect the mean absolute color change of each dye on the CSA. The CSA’s color change is obvious for high concentration amines, but barely visible for TMA concentrations lower than 8 ppm, which means some more complicated methods rather than naked-eye discrimination are necessary to improve the CSA’s gas detection capability.

**Figure 5.**
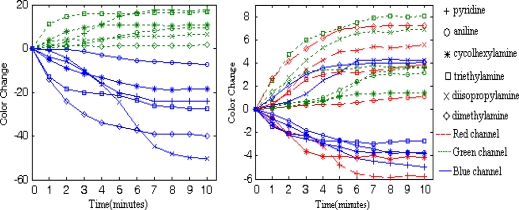
Representative responses for dye H2OEP (left) and dye CoTPP (right), showing the magnitudes of red, green and blue color changes over the course of 10 minutes exposure to the six amine headspace vapors. The response curves on the red channel for H2OEP gather in the zero neighboring area with a biggest magnitude of 5

**Figure 6.**
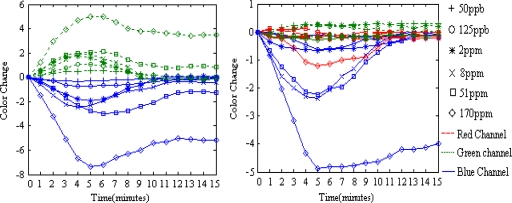
Representative responses for dye H2OEP (left) and dye CoTPP (right), showing the magnitudes of red, green and blue color changes over the course of 5 minutes exposure to the six TMA concentrations and followed by 10 minutes exposure to N_2_ for reproducibility test. The response curves on the red channel for H2OEP gather in the zero neighboring area with a biggest magnitude of 0.7.

Representative color responses for two dye spots to all the analytes are shown in Figures 5 and 6. The color change amplitude of the red, green and blue color channels of the two dye spots are shown over the exposure time to different analyses. Data are plotted after the following processes:

**Image simplification:** Locate each dye spot on the CSA and sample 250 pixels in the center of the dye spot to determine the average red, green and blue values of each dye spot, thus simplifying a CSA image to 18 color values.

**Color sequence filtering:** According to the experimental setup and image simplifying process, for any color channel on a CSA, we obtain color sequences of 15 points for qualitative discrimination and 20 points for quantitative detection. The color sequences are filtered according to Equation (1) before further processes, *C^n^* being certain color channel of the nth dye spot on the CSA:

(1) $$C_{n}(t_{k})=(\sum_{i=k-2}^{k+2}C_{n}(t_{i})-\text{max}(C_{n}(t_{i}),i\in (k-2,k+2))-\text{min}(C_{n}(t_{i}),i\in (k-2,k+2)))/3$$

**Baseline manipulation:** The solution casting method of CSA fabrication cannot ensure the color consistency of the same dye on different CSAs. As for the CSAs used in this experiment, these kinds of color differences are within six color units. We consider the color differences as sensor drifts and perform a differential baseline manipulation to reduce the drift effects [17]:

(2) $$\mathrm{cl}\text{Re}\mathrm{sp}(C_{n},t_{k})=C_{n}(t_{k})-C_{n}(t_{0})$$

where *t_0_* is the image sampling time immediately before the CSA’s exposure to analytes, and *cl*Re*sp*(*t_k_*) is the CSA’s color response at time *t_k_* on any one of the 18 color channels counting from the exposure start time by minute.

For H2OEP, the color changes occur mainly in the green and blue color channels, where an increment of green color values and a decrease of blue color values can always be observed for each analyte.

The performance is quite different for dye CoTPP. When coordinated with amine molecules, a ‘core expansion’ effect on the dye’s molecular ring structure ocurrs [18], which leads to a red shift of the dye’s absorption spectrum, and/or an an energy perturbation effect on the dye’s frontier molecular orbits [19], which leads to a blue shift of the dye’s absorption spectrum determined mainly by the dye’s central metal ion. For H2OEP, only the ‘core expansion’ effect takes place upon interaction with the analytes. As a result, the color change direction in a certain color channel is the same and the color change amplitude is relatively high; For CoTPP, both effects takes place but either the ‘core expansion’ effect or the energy perturbation effect may dominate upon interaction with amines, which is determined by the electronic properties of the amine molecule in question. As a result, the color change directions in a certain color channel may be different for different amine analytes and the color change amplitude is relatively low.

**Figure 7.**
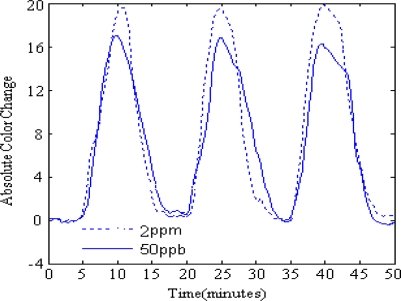
Repeating cycling of TMA exposure of CSA from N_2_ to 2 ppm (dotted line) and 50 ppb (solid line), respectively. The color change value is the sum of the absolute color changes in all the 18 color channels on the CSA.

On the CSA, a given dye shows different transient and steady color response to different analytes, while a given analyte leads to different color change progress for different sensitive dyes, which lead to effective color change patterns suitable for analyte discrimination. The CSA shows different dynamic color change progress for different TMA concentrations. At the response stage, The CSA’s color response to TMA concentrations of ppb level takes place slowly and the color change can be measured until the 2^nd^ minute of exposure, after that the color change soon reaches saturate in no more than 2 minutes; meanwhile, its color response to TMA concentrations of ppm level takes place almost immediately once the exposure begins. While the color changes for TMA concentrations of 2 ppm and 8 ppm can reach saturation after 4 minutes’ exposure, the color changes for TMA concentration of 51 ppm and 170 ppm may continue if the exposure lasts longer than 5 minutes. Similar phenomena can be observed at the recovery stage. When washed with a N_2_ stream, CSAs contacted with ppb level TMA concentrations don’t show any color change in the first 3 minutes and soon change color back to their initial state in no more than 3 minutes after that; CSAs contacted with ppm level TMA concentrations show color changes almost immediately once the N_2_ begins to flow, but only those CSAs that reacted with low concentrations can restore their color in ten minutes, while high concentrations of TMA can lead to non-reproducibility of the color response for CSAs. As shown in Figure 6, for 170 ppm and 51 ppm TMA, the dye colors do not restore to the initial values after being washed with a N_2_ stream for 10 minutes. Reproducibility is observed with TMA concentrations below 8 ppm (shown in Figure 7), as the CSA reproducibly cycles between N_2_ and some TMA concentration. Even for the low concentration of 50 ppb, the CSA’s color response is pronounced, which means that the CSA’s LOD for TMA can be very low.

### 3.2. Response Feature Extraction

RGB color space is the most widely used device dependent color space, but is not necessarily the best color space for color pattern recognition [16,20]. We report here a feature extraction method by mixing several different color spaces into a mixed color space and then extracting those color channels with the best analytes discriminant capability for the CSA to compose a fusional color space for further statistical analysis, such as PCA or HCA. The Canon SX100 supports the sRGB color standard, under which a RGB image can be easily transformed into other common color spaces. Images sampled in the RGB color space are transformed into color spaces of XYZ, L*a*b*, HSV, YIQ, and I1I2I3 [21], respectively. The six color spaces are then mixed together to compose a mixed color space, named as “Mix”, with 18 color channels [RGBXYZL*a*b*HSVyIQI1I2I3]. In the mixed color space, the color channel Y from YIQ is written as y to discriminate the Y from XYZ. As illustrated in Figures 5 and 6, the dyes’ transient responses to different analytes provide important discriminant information. A time-stacked feature vector, tFv, from any one trial is given in Equation (3), using images collected in the first five minutes after exposure to analytes:

(3) $$\begin{array}{rr} \text{tFv} & =[\text{clResp}(R1,t1)\text{clResp}\left(R2,t1\right)\;\ldots \text{clResp}\left(R6,t1\right)\ldots \\ & \text{clResp}\left(I31,t1\right)\text{clResp}\left(I32,t1\right)\ldots \text{clResp}\left(I36,t1\right)\ldots \\ & \text{clResp}\left(R1,t2\right)\text{clResp}\left(R2,t2\right)\ldots \text{clResp}\left(R6,t2\right)\ldots \\ & \text{clResp}\left(I31,t2\right)\text{clResp}\left(I32,t2\right)\ldots \text{clResp}\left(I36,t2\right)\ldots \\ & \text{clResp}\left(R1,t5\right)\;\text{c1Resp}\left(R2,t5\right)\ldots \text{clResp}\left(R6,t5\right)\ldots \\ & \text{clResp}\left(I31,t5\right)\text{clResp}\left(I32,t5\right)\ldots \text{clResp}\left(I36,t5\right)] \end{array}$$

For a CSA with six dye spots and a mixed color space with n color channels, the five points time-stacked feature vector tFv has 30 × n dimensions. For the 60 trials, a feature matrix tFm of 60 × 30 × n dimensions can be obtained. All column vectors of matrix tFm were normalized at first for further processing, but as illustrated in Figures 5 and 6, some color channels, such as the red channel of the sixth dye spot, are less important for anaylte discrimination. We perform a joint search algorithm of sequential forward selection and sequential backward selection (SFS&SBS) to pick out 18 most classificationally valuable color channels based on a linear discriminant criteria (LDC) given in Equation (4):

(4) $$\mathrm{Jabc}=\frac{\frac{1}{N}\sum_{i=1}^{N}(m_{i}-m){\left(m_{i}-m\right)}^{T}}{\frac{1}{N}\sum_{i=1}^{N}\frac{1}{M}\sum_{j=1}^{M}(x_{\mathrm{ij}}-m_{i}){\left(x_{\mathrm{ij}}-m_{i}\right)}^{T}}$$

In Equation (4), abc is some sub matrix of tFm obtained by extracting columns of given color channels, namely the feature matrix of color space abc; x is the row vector of matrix abc; N is the class number, equals to 12 for the 12 analytes; M is the number of observation samples in each class which is equal to 5 since we did five replicate trials for each analyte; mi is the sample center of the ith class by averaging all the class vectors; m is the center of all the observation samples in color space abc. A bigger Jabc means better identification ability in abc color space.

For the Mix color space, the CSA has 108 different color channels. The SFS&SBS search algorithm select the 18 color channels through following steps: assuming that K channels are already picked out, first we execute the SFS algorithm to pick out another four channels in the remained 108-K channels which makes *J*_*K*+4_ the biggest; then perform SBS algorithm to eliminate two color channels in the K + 4 which makes *J*_*K*+4−2_ the biggest. The color channels selected by the search algorithm are H6, Z1, b*6, Z2, Y3, I26, B6, Z6, L6, G3, S6, Y6, G6, B1, L3, B2, Q6, and y6, which compose the fusional color space. Vectors in the fusional color space, Fus, or other common color spaces, have 90 feature elements. The data is further processed using PCA to remove redundant feature components and reduce the interferences within. A comparison of the CSA’s analytes identification ability in different color spaces is given in Table 1, where cmpr means the percentage of information compressed by PCA. In Table 1, the row data is the LDC scores of different color spaces at certain PCA compression rate, which can tell the difference of classification ability among all the color spaces. It could be noted that for any of the compression rate, the fusional color space scores the highest and shows the best sample classification potential. The column data of Table 1 is the LDC scores of given color spaces at different PCA compression rate, which can tell the effect of PCA compression on the sample classification ability on a color space. For any of the color spaces, the LDC score increases monotonically with the increment of PCA compression rate, which means that PCA transformation does increase the identification performances for any color space.

### 3.3. Grouping of the Analytes

**Figure 8.**
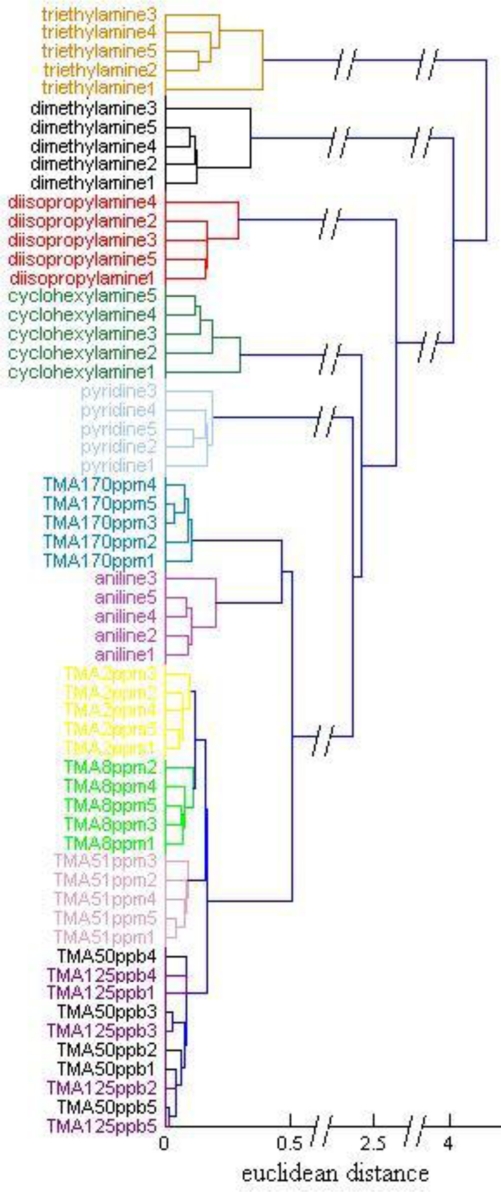
HCA dendrogram for 12 amine analytes using euclidean distance and single linkage with the Matlab statistic tool box.

A combination of PCA and HCA were used in the Fus color space to group the 60 trial samples. The feature vectors in Fus space were at first compressed to six dimensions by PCA, then the sixty 6-dimentional sample vectors were grouped by HCA (from the Matlab statistic toolbox), where the distance between vectors was set as ‘euclidean’ and the linkage ways ‘single’. The HCA dendrogram is shown in Figure 8, where the six amines for qualitative identification and four of the TMA concentrations at ppm level are correctly grouped. While TMA concentration of ppb level are correctly discriminated from other samples, the concentrations of 125 ppb and 50 ppb are indistinguishable from each other. The good performance of sample grouping of the sensor is not strange since all the feature vectors are picked out from a large family of possible feature vectors according to the linear discriminant criteria (LDC). Since we didn’t train a strict classification model for the 60 samples, the performance of the feature extraction method introduced here in gas classification need to be verified in the next work. The CSA’s concentration resolution is determined mainly by the imaging devices. Image sensors with higher pixel resolution and longer data storing bits may improve the CSA’s performance. The six TMA concentrations are clustered in a large group with aniline mixed in, meaning that the CSA has the potential for identifying amines and determining their concentrations simultaneously if a more sophisticated algorithm is conducted [22]. Furthermore, aromatic amines (aniline and pyridine), an aliphatic cyclic amine (cyclohexylamine), and branched amines (diisopropylamine, dimetylamine and triethylamine) are added to the group sequentially, showing that the CSA can to some degree recognize the molecular structure of analytes, according to their coordination ability to the sensitive dyes on the CSA. In fact, we can see all the analytes as TMA with different concentrations, and the lower concentrations lead to weaker response effects. Since the TMA also has branched molecular structure, we can assume that the three kinds of branched amines with saturates concentrations are similar to TMA of saturates concentration, which is thousands of ppm under the experimental conditions. The aromatic amines can interact with the dyes on CSA with Pi-Pi interaction besides coordination. Pi-Pi interaction is a phenomenon in organic chemistry that affects aromatic compounds and functional groups. As shown in Figure 1, all the sensitive dyes have large flat aromatic ring surfaces. The Pi electronic orbits between the surfaces of the sensitive dyes and the aromatic ring of aniline or pyridine may interact with each other, which tends to arrange them like stack of coins. The Pi-Pi interaction changes the electronic cloud distribution of the polymer, which may obviously weaken the effect of coordination and make aromatic amines like TMA of very low concentrations more. The aliphatic cyclic amines generate bigger steric hinderance effect on coordinating to the sensitive dyes than branched amines do, which makes them reacting with the dyes by weaker bonding power and may be seen as lower TMA concentrations than the branched amines.

## 4. Conclusions

We here present a promising colorimetric sensor for qualitative identification and quantitative detection of volatile amines. The dyes are carefully chosen based upon the properties of the amine analytes to achieve high selectivity and sensitivity. CSA’s color changes in different color spaces show different amine identification ability and the best performance is achieved in the fusional color space. The format of the sensor system can improve portability by replacing the host PC with an embedded processing module. For further development, imaging devices with higher performance and CSAs with more dye spots are expected to boost the sensor’s comprehensive performance. More reasonable and flexible combination of dyes on sensitive arrays, more pertinent feature extraction and more sophisticated pattern recognition algorithm should improve the sensor versatility in various applications.

## Figures and Tables

**Table 1.** LDC scores of all the color spaces under different PCA compression rate.

**cmpr(%)**	**JRGB**	**JXYZ**	JL*a*b*	**JYIQ**	**JHSV**	**JI1I2I3**	**JMix**	**JFus**
0	22.984	47.833	18.974	26.609	10.897	26.477	21.731	238.68
2	38.07	110.92	25.964	48.996	13.673	42.645	35.321	434.73
4	59.058	123.78	41.467	83.127	16.981	79.431	73.28	476.62
6	119.55	147.35	70.534	138.83	21.45	151.77	120.34	515.95
8	119.55	161.01	122.54	138.83	27.287	151.77	158.92	515.95
10	161.37	161.01	122.54	138.83	36.217	151.77	190.17	515.95

## Abbreviations:

CSA: Colorimetric sensor array
TMA: Trimethylamine
LDC: Linear discriminant criteria
PCA: Principle component analysis
HCA: Hierarchical cluster analysis
LOD: Limitation of detection
TPP: Tetraphenylporphine
OEP: Octaethylporphine
Pc: Phthalocyanine
SFS&SBS: a joint search algorithm of sequential forward selection and sequential backward selection
